# Proteomic insight into arabinogalactan utilization by particle-associated *Maribacter* sp. MAR_2009_72

**DOI:** 10.1093/femsec/fiae045

**Published:** 2024-04-03

**Authors:** Saskia Kalenborn, Daniela Zühlke, Katharina Riedel, Rudolf I Amann, Jens Harder

**Affiliations:** Department of Molecular Ecology, Max Planck Institute for Marine Microbiology, Celsiusstr. 1, D-28359 Bremen, Germany; Department for Microbial Physiology and Molecular Biology, University of Greifswald, Felix-Hausdorff-Str. 8, D-17489 Greifswald, Germany; Department for Microbial Physiology and Molecular Biology, University of Greifswald, Felix-Hausdorff-Str. 8, D-17489 Greifswald, Germany; Department of Molecular Ecology, Max Planck Institute for Marine Microbiology, Celsiusstr. 1, D-28359 Bremen, Germany; Department of Molecular Ecology, Max Planck Institute for Marine Microbiology, Celsiusstr. 1, D-28359 Bremen, Germany

**Keywords:** alpha-l-arabinofuranosidase, *Flavobacteriia*, GH43, marine bacteria, proteomics, unsaturated rhamnogalacturonylhydrolase

## Abstract

Arabinose and galactose are major, rapidly metabolized components of marine particulate and dissolved organic matter. In this study, we observed for the first time large microbiomes for the degradation of arabinogalactan and report a detailed investigation of arabinogalactan utilization by the flavobacterium *Maribacter* sp. MAR_2009_72. Cellular extracts hydrolysed arabinogalactan *in vitro*. Comparative proteomic analyses of cells grown on arabinogalactan, arabinose, galactose, and glucose revealed the expression of specific proteins in the presence of arabinogalactan, mainly glycoside hydrolases (GH). Extracellular glycan hydrolysis involved five alpha-l-arabinofuranosidases affiliating with glycoside hydrolase families 43 and 51, four unsaturated rhamnogalacturonylhydrolases (GH105) and a protein with a glycoside hydrolase family-like domain. We detected expression of three induced TonB-dependent SusC/D transporter systems, one SusC, and nine glycoside hydrolases with a predicted periplasmatic location. These are affiliated with the families GH3, GH10, GH29, GH31, GH67, GH78, and GH115. The genes are located outside of and within canonical polysaccharide utilization loci classified as specific for arabinogalactan, for galactose-containing glycans, and for arabinose-containing glycans. The breadth of enzymatic functions expressed in *Maribacter* sp. MAR_2009_72 as response to arabinogalactan from the terrestrial plant larch suggests that *Flavobacteriia* are main catalysts of the rapid turnover of arabinogalactans in the marine environment.

## Teaser

The marine particle-associated flavobacterium *Maribacter* degrades larch wood arabinogalactan with multiple CAZymes located in three polysaccharide utilization loci and outside according to proteomic investigation.

## Introduction

Marine environments contain many different polysaccharides as dissolved organic matter (DOM) or in particulate organic matter (POM). These are a vital carbon source for microorganisms, released from algae as exudates or during lysis by zooplankton predation or viral infection. Monosaccharide analysis of planktonic biomass from the North Sea revealed already in 1982 a dominance of glucose followed by arabinose, galactose, and mannose (Ittekkot et al. 1982, Urbani et al. 2005, Alderkamp et al. 2007, Scholz and Liebezeit 2013, Huang et al. 2021). These monomers are the building blocks of algal polysaccharides: the abundant beta-homoglycans laminarin, cellulose, and xylan are often complemented with species-specific glycans such as agar, alginate, carrageenan, fucoidan, mannan, pectin, porphyran, and ulvan. The degradation of these glycans has been studied intensively in marine systems, however, details for arabinogalactan are missing (Bäumgen et al. 2021). Recently arabinogalactan was detected in the high molecular weight dissolved organic matter (HMWDOM) and POM fraction using monoclonal antibodies during the algal spring bloom in the North Sea (Vidal-Melgosa et al. 2021). This coincides with the high arabinose and galactose content of *Phaeocystis* spp., a haptophyte blooming in the North Sea (Alderkamp et al. 2007, Sato et al. 2018). The antibody-based quantification also showed a decrease in arabinogalactan content towards the end of the spring bloom, suggesting a fast turnover of the compound—contrasting with the accumulation of fucose-containing sulfated polysaccharides (Vidal-Melgosa et al. 2021). The major source of arabinose and galactose in algae are likely arabinogalactan proteins, which anchor polysaccharide cell walls in the outer membrane of plants and algae (Silva et al. 2020, Leszczuk et al. 2023). The model compound for arabinogalactan type II is arabinogalactan from larch wood. It contains d-galactose and l-arabinose in a 6:1 molar ratio as well as traces of rhamnose, fucose, mannose, xylose, and d-glucuronic acid (Fujita et al. 2019, Villa-Rivera et al. 2021, Leszczuk et al. 2023). Type II arabinogalactans have a complex backbone structure consisting of β-1,3-linked galactan backbone with β-1,6-linked galactan side chains (Kelly 1999, Wang and LaPointe 2020). Type I has a β-1,4-linked galactan backbone, whereby C3 can be linked with l-arabinofuranose (Hinz et al. 2005).

Plant arabinogalactan is degraded by aerobic bacteria and fungi as well as by anaerobic fermenting bacteria in gut systems, including *Bifidobacterium* and *Bacteroidetes* (Shulami et al. 2011, Ndeh et al. 2017, Cartmell et al. 2018, Luis et al. 2018, Wang and LaPointe 2020, Sasaki et al. 2021). The latter phylum encompasses also aerobic *Flavobacteriia* that have been identified as specialists for polysaccharide degradation in marine systems (Sidhu et al. 2023). For this first study on the degradation of arabinogalactan by marine microorganisms, we selected a flavobacterial strain with a published genome and a particle-associated lifestyle, *Maribacter* sp. MAR_2009_72 (Kappelmann et al. 2018, Heins et al. 2021a). Strains of the genus *Maribacter* are rarely isolated from sea water, but they are more abundant in particle fractions (Nedashkovskaya et al. 2004, Heins and Harder 2023, Lu et al. 2023, Sidhu et al. 2023). Abundances of up to 4% were detected in the oxic surface layer of sandy sediments (Probandt et al. 2018, Miksch et al. 2021). Even higher abundances were observed in micro- and macroalgae phycosphere populations (Heins et al. 2021b, Lu et al. 2023). This makes *Maribacter* strains ideal candidates for studying the degradation of algal cell wall polysaccharides.

The uptake and degradation of polysaccharides in *Bacteroidetes* is often encoded in polysaccharide utilization loci (PULs). The first PUL was described for *Bacteroides thetaiotaomicron* for starch utilization (Shipman et al. 2000). Polysaccharide utilization starts with the extracellular hydrolysis of polysaccharides into oligosaccharides on the surface of the cell. The oligosaccharides are transported into the periplasm via the SusC/D transport system, which is energized by a proton gradient via an ExbB/D-TonB system in the cytoplasmic membrane and by a domain in the periplasm to open the β-barrel channel of SusC for the transport (Noinaj et al. 2010). The hydrolysis of polysaccharides is achieved by glycoside hydrolases (GH), glycoside transferases, polysaccharide lyases, and carbohydrate esterases with a high specificity, sometimes assisted by carbohydrate binding modules. These five groups of proteins are classified as carbohydrate active enzymes (CAZymes) (Bäumgen et al. 2021, Drula et al. 2022). For the degradation of arabinogalactan from larch wood, PULs were so far characterized for gut bacteria including *Bifidobacterium longum* ssp. *longum* NCC2705, *Bacteroides caccae* ATCC 43185, and *Bacteroides thetaiotaomicron* (Ndeh et al. 2017, Cartmell et al. 2018, Luis et al. 2018, Wang and LaPointe 2020). Here, we analyzed *Maribacter* sp. MAR_2009_72 proteomes using cells grown on arabinogalactan, arabinose, galactose, and glucose. Those proteomes were compared to identify the proteins induced by arabinogalactan. This study expands a recent *in silico* study that did not report on arabinogalactan-specific PULs (Kappelmann et al. 2018) and provides experimental observations for a better interpretation of marine metagenomes.

## Material and methods

### Growth experiments

*Maribacter* sp. MAR_2009_72 (DSM 29384), originally isolated from a phytoplankton catch in the Wadden Sea near the island Sylt, Germany, was revived from glycerol stocks that had been preserved in the laboratory since the initial isolation (Hahnke and Harder 2013). The strain was grown in the liquid medium HaHa_100 V with 0.3 g/l of casamino acids as the sole carbon source (Hahnke et al. 2015). This limited growth to an optical density (OD) at 600 nm below 0.2. Growth beyond an OD of 0.3 was achieved by adding 2 g/l of a carbohydrate source, here arabinose, galactose, glucose (Sigma Aldrich/Merck KGaA, Darmstadt, Germany), and larch arabinogalactan (The Dairy School, Auchincruive, Scotland). The supplier of arabinogalactan had specified the monosaccharide composition as 81% galactose, 14% arabinose, and 5% other, whereby the other fraction was not defined. For proteomics, three cultures of 50 ml were inoculated with 0.4% v/v of a pregrown culture in the same medium and incubated at room temperature at 110 r/m. A fourth culture per substrate was maintained to monitor bacterial growth by measuring OD at 600 nm beyond the harvest point. Cells were harvested at an OD of 0.25. Cells were pelleted by centrifugation in 50 ml tubes with 3080 × *g* for 30 min at 4°C. Pellets were resuspended in 1 ml medium and centrifuged in 1.5 ml tubes at 15870 × *g* for 15 min at 4°C. The wet biomass was weighed and stored at −20°C.

For microbiome size determinations, colony-forming units (CFU) were determined with 4 g/l larch wood arabinogalactan as organic carbon source on marine plates (Hahnke and Harder, 2013), using 4 g/l glucose or ZoBell’s 2216 marine agar plates as reference. Inoculation of serial diluted sea or sediment pore water was performed with a 96 pin-holder. Inoculations were at room temperature. Partial 16S rRNA gene sequences of strains were obtained by colony PCR and Sanger sequencing (Hahnke and Harder 2013). Partial 16S rRNA gene sequences have been deposited at GenBank under the accession numbers PP600029 to PP600099.

### Protein preparation and mass spectrometry

Proteins were extracted from cells using a bead-beating method following the protocol by Schultz et al. (2020). A pellet of wet weight ranging from 20 to 200 mg was disrupted using 0.25 ml glass beads in 500 µl of lysis buffer. The protein content was quantified using the Roti Nanoquant assay (Carl Roth, Karlsruhe, Germany). For protein purification on denaturing polyacrylamide gels (SDS-PAGE), 50 µg of protein was combined with 10 µl of 4x SDS buffer [composed of 20% glycerol, 100 mM Tris/HCl, 10% (w/v) SDS, 5% β-mercaptoethanol, 0.8% bromophenol blue, pH 6.8] and loaded onto Tris-glycine-extended precast 4%–20% gels (Bio-Rad, Neuried, Germany). Electrophoresis was conducted at 150 V for 8 min. Subsequently, the gel was fixed in a solution of 10% v/v acetic acid and 40% v/v ethanol for 30 min, stained with Brilliant Blue G250 Coomassie, and the desired protein band was excised. The proteins were extracted from the gel in one piece and then washed with a solution of 50 mM ammonium bicarbonate in 30% v/v acetonitrile. The gel pieces were dried using a SpeedVac (Eppendorf, Hamburg, Germany), and then rehydrated with 2 ng/µl trypsin (sequencing grade trypsin, Promega, USA). After a 15-min incubation at room temperature, excess liquid was removed, and the samples were digested overnight at 37°C. Following digestion, the gel pieces were covered with water suitable for mass spectrometry (MS), and peptides were eluted using ultrasonication. The peptides were subsequently desalted using Pierce™ C18 Spin Tips (Thermo Fisher, Schwerte, Germany) in accordance with the manufacturer’s guidelines. The eluted peptides were dried using a SpeedVac and stored at −20°C. For MS analysis, the samples were thawed and reconstituted in 10 µl of Buffer A (99.9% acetonitrile + 0.1% acetic acid).

Tryptic peptides of *Maribacter* sp. MAR_2009_72 were analyzed using an EASYnLC 1200 system coupled to a Q Exactive HF mass spectrometer (Thermo Fisher Scientific, located in Waltham, USA). Peptides were loaded onto a custom-packed analytical column containing 3 µm C18 particles (Dr. Maisch GmbH, Ammerbuch, Germany). The loading was performed using buffer A (0.1% acetic acid) at a flow rate of 2 µl/min. Peptide separation was achieved through an 85-min binary gradient, transitioning from 4% to 50% buffer B, composed of 0.1% acetic acid in acetonitrile, at a flow rate of 300 nl/min. Samples were measured in parallel mode; survey scans in the Orbitrap were recorded with a resolution of 60 000 with a m/z range of 333 to 1650. The 15 most intense peaks per scan were selected for fragmentation. Precursor ions were dynamically excluded from fragmentation for 30 s. Single-charged ions as well as ions with unknown charge state were rejected. Internal lock mass calibration was applied (lock mass 445.12003 Da).

The MS files were analyzed in MaxQuant version 2.2.0.0 in the standard settings against the strain specific protein database downloaded from NCBI: Maribacter sp. MAR_2009_72 NZ_VISB01000001.1 (27 September 2022) and common laboratory contaminants (Tyanova and Cox 2018, Sayers et al. 2022). Statistical analysis was performed in Perseus version 2.0.7.0 (Tyanova and Cox 2018). Proteins were recognized as being expressed when they had label free quantification intensities (LFQ) intensities in one out of three biological replicates.

### Bioinformatic analyses

Protein annotation was refined using several databases. CAZymes were considered to be identified, if two out of three search algorithms in dbCAN3 were positive (Zheng et al. 2023). The conserved domain database (CDD) (Lu et al. 2019), the SulfAtlas web interface (Stam et al. 2022), InterPro (Paysan-Lafosse et al. 2022), PULDB (Terrapon et al. 2018), deepTMHMM (Hallgren et al. 2022), SignalP (Teufel et al. 2022), Blastkoala (Kanehisa et al. 2016), and UniProt (The UniProt 2023) provided additional information.

The search pattern used in PULDB were the following for PUL 1: GH10+GH43_1+GH67; for PUL 7: GH43_18+GH78+GH105+GH105+GH115; and for PUL 8: GH43_19+GH43_34+GH51+GH105+GH105.

For the visualization of the data the following programs and packages were used: R version 4.3.2 (R Core Team 2023), ggplot2 (Wickham 2016), gggenes (Wilkins 2023), and Proksee (Grant et al. 2023).

The MS proteomics data have been deposited to the ProteomeXchange Consortium via the PRIDE (Perez-Riverol et al. 2022) partner repository with the dataset identifier PXD049074 and 10.6019/PXD049074.

## Results

### Growth on arabinogalactan

**Figure 1.**
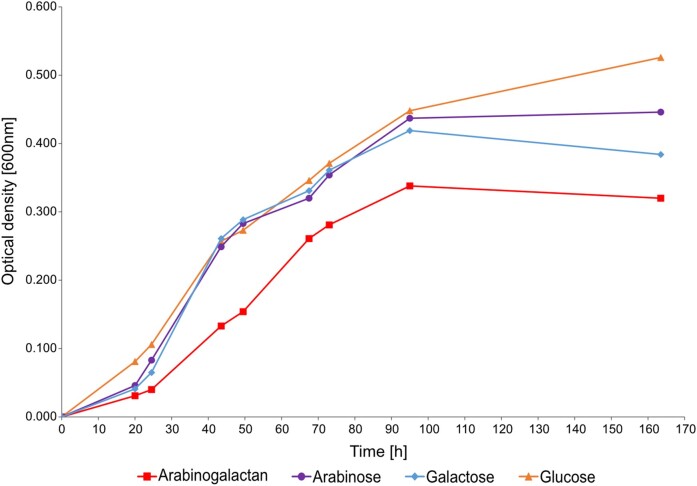
Growth curve of *Maribacter* sp. MAR_2009_72 in presence of four different carbon sources; arabinogalactan, arabinose, galactose, and glucose. MAR_2009_72 was grown in 50 ml of modified HaHA100V with 2 g/l of the respective carbon source at room temperature at 110 r/m. The OD was measured at 600 nm.

*Maribacter* sp. MAR_2009_72 grew in presence of larch wood arabinogalactan to a maximum OD of 0.338 and at a maximum growth rate *µ* = 0.06 h^−1^ (Fig. 1). When 2 g/l of galactose or arabinose were provided in the medium a maximum OD of 0.419 and 0.446 was measured with respective growth rates of 0.07 h^−1^ and 0.06 h^−1^. Glucose supported the largest biomass formation, with an OD of 0.526 and *µ* = 0.05 h^−1^. The arabinogalactan cultures required more time to enter the exponential growth phase than the cultures with monosaccharides as substrates. The physiological reaction was confirmed *in vitro*. Soluble cell extracts hydrolysed larch wood arabinogalactan to an abundant molecule with the size of a hexose (galactose) and a minor molecule with the size of a pentose (arabinose) in fluorophore-assisted carbohydrate electrophoresis gels (Fig. S1, Supporting Information).

### Protein expression in *Maribacter* sp. MAR_2009_72

**Figure 2.**
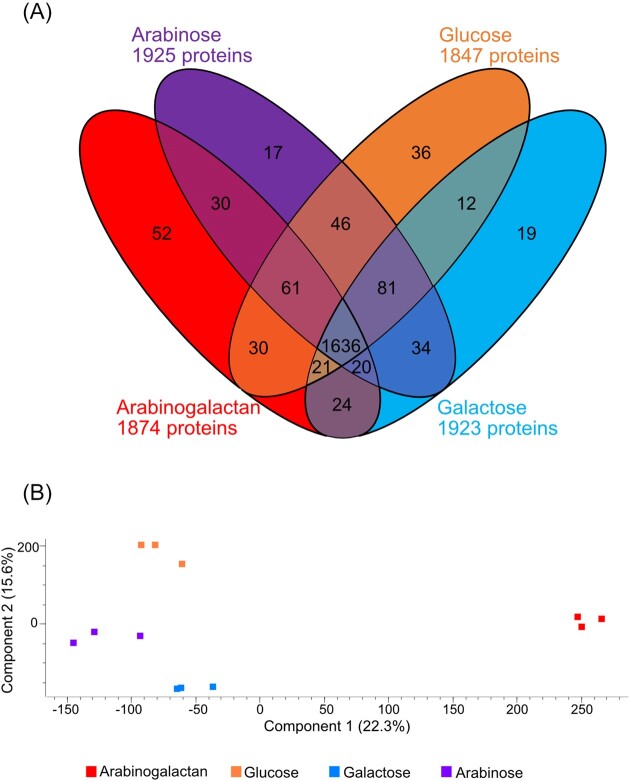
Comparison of the number of detected proteins in arabinogalactan, arabinose, galactose, and glucose. (A) Venn diagram showing the overlap of detected proteins in at least one of three biological replicates. (B) Principal component analysis shows the differences between the expression intensities of the four proteomes of MAR_2009_72.

The comparative proteomic analysis was based on glucose as reference against arabinose, galactose, and arabinogalactan. We identified 1874 proteins in the arabinogalactan proteome (Fig. 2A). Overall, these four conditions shared 1636 proteins. Only a small number of proteins were found to be unique to a particular growth condition. The glucose proteome had 36 unique proteins, the arabinose proteome 17 proteins, and the galactose proteome 19 proteins. Arabinogalactan had 52 unique proteins. We used the expression data, here label free quantification intensities (LFQ), to visualize the difference between the four conditions in a principal component analysis (PCA) (Fig. 2B). The PCA plot indicated that the arabinogalactan proteome had the most contrasting expression pattern. The PCA analysis documented that the differences between monosaccharide proteomes were less pronounced than to the arabinogalactan proteome.

**Figure 3.**
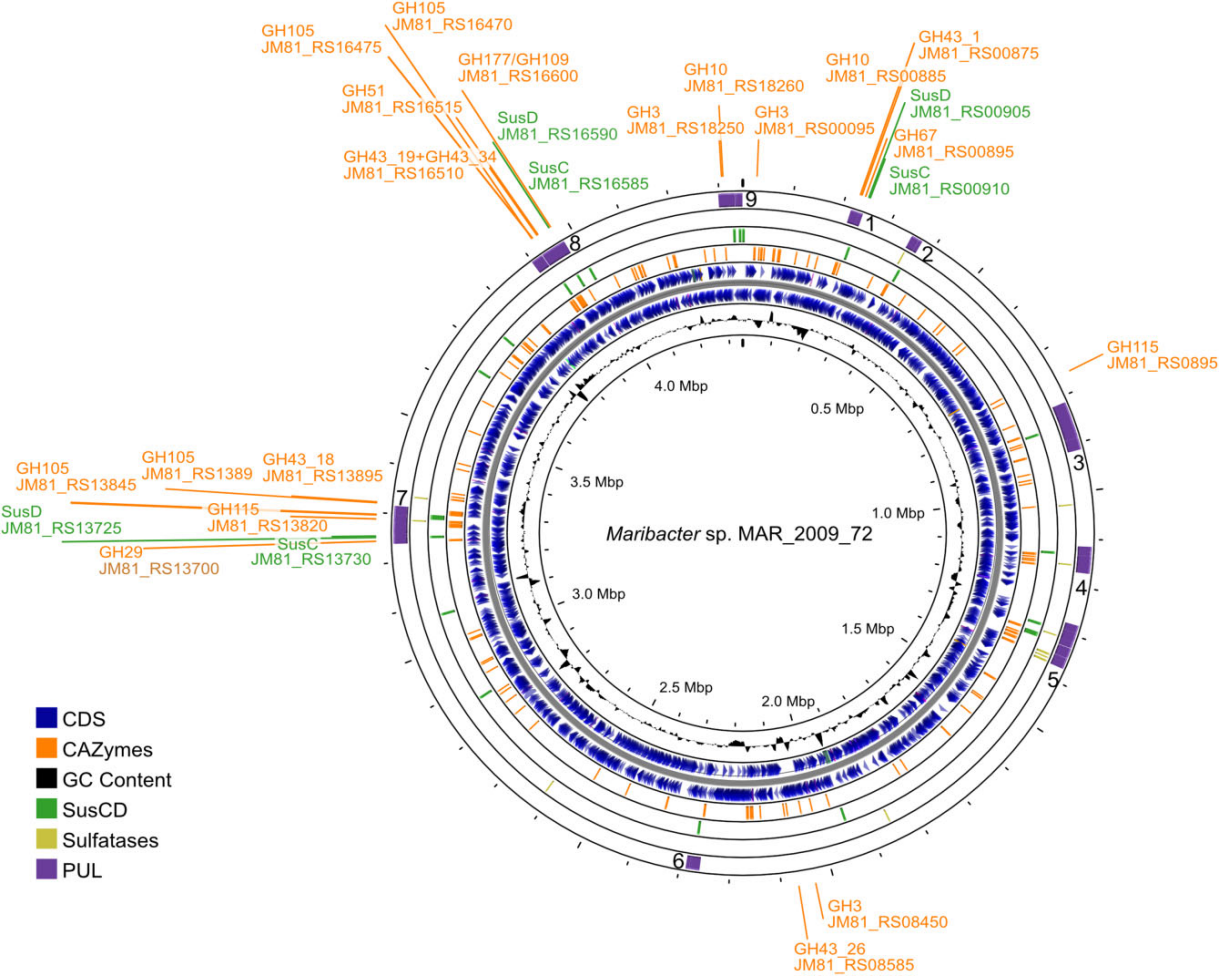
Full genome overview of *Maribacter* sp. MAR_2009_72 showcasing the GC content (ring one (most inner ring)), all annotated coding genes (CDS, ring two and three) in forward and reverse direction, CAZymes identified by dbCAN3 (ring four), SusC/D (ring five), sulfatases (ring six), and PULs (ring seven). Furthermore, we highlighted CAZymes and SusC/Ds that might be important for arabinogalactan utilization.

*Maribacter* sp. MAR_2009_72 has a genome of 4.35 Mb encoding 3635 proteins (Fig. 3). Nine PULs contain one or several SusC/D transporter and neighboring CAZymes. We labelled the PULs based on the arrangement in the genome, with PUL 1 being closest to the origin of replication (Table S1, Supporting Information). The expression values revealed a proteomic response to arabinogalactan in PUL 1, 7, and 8 and outside of PULs.

**Figure 4.**
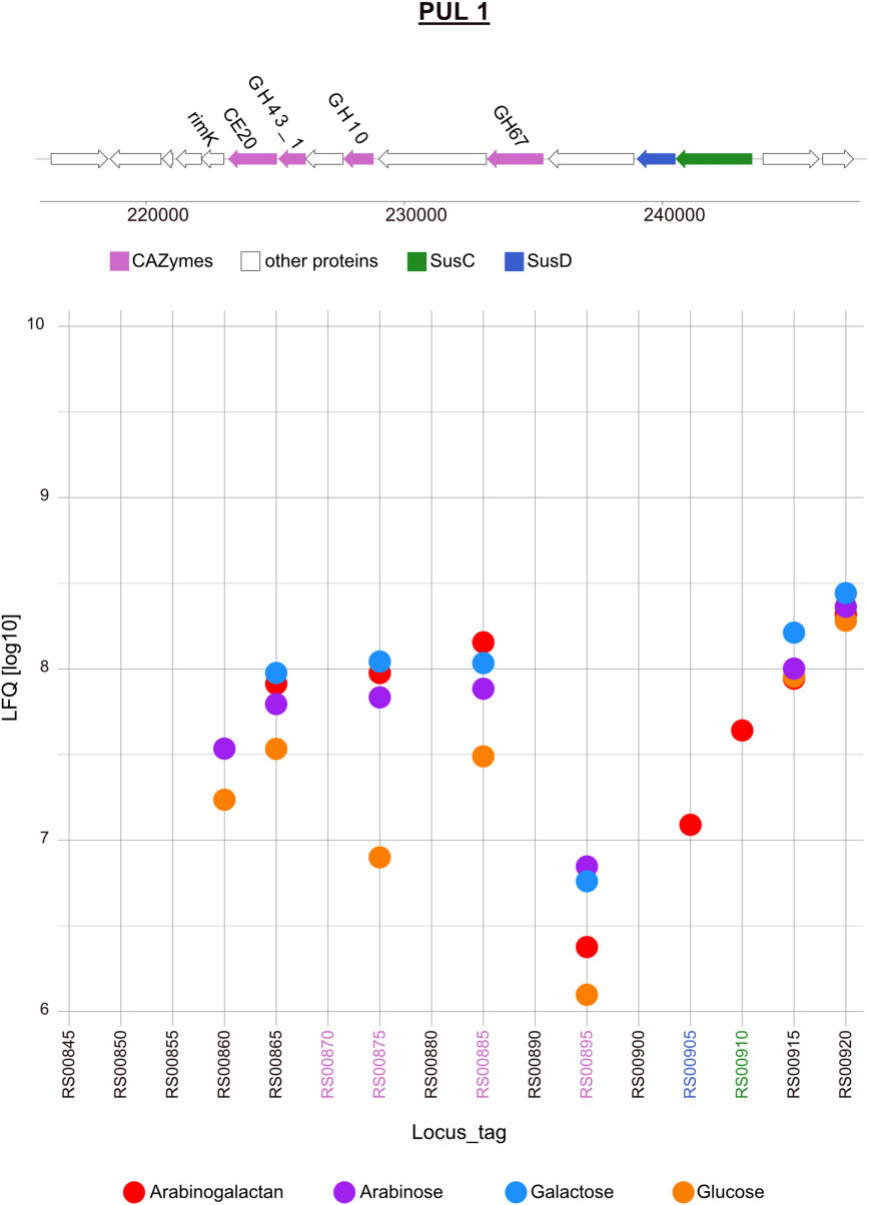
Gene organization and expression of polysaccharide utilization locus 1 of *Maribacter* sp. MAR_2009_72 grown in the presence of arabinogalactan, arabinose, galactose, and glucose. Expression intensities in the plot are the mean values of three biological replicates of each condition shown in LFQ values [log10].

PUL 1 encodes 13 proteins of which three out of four CAZymes and one SusC/D pair were expressed in arabinogalactan grown cells (Fig. 4). The SusC/D pair (JM81_RS00910 and JM81_RS00905) was only expressed in the arabinogalactan proteome. Four other proteins were clearly induced by arabinogalactan, arabinose, and galactose. The α-l-arabinofuranosidase GH43_1 (JM81_RS00875) was 10-fold induced relative to the glucose proteome. A GH10, an endo-β-1,4-xylanase, showed a similar expression pattern with a 5-fold difference to glucose. The third enzyme was a GH67, an α-glucuronidase, which had the strongest induction in arabinose and galactose proteomes. The fourth induced protein of the operon with an expression in the arabinogalactan proteome affiliated to the superfamily of protein or cofactor modifying RimK-type glutamate ligases with an ATP-grasp binding domain (JM81_RS00865).

**Figure 5.**
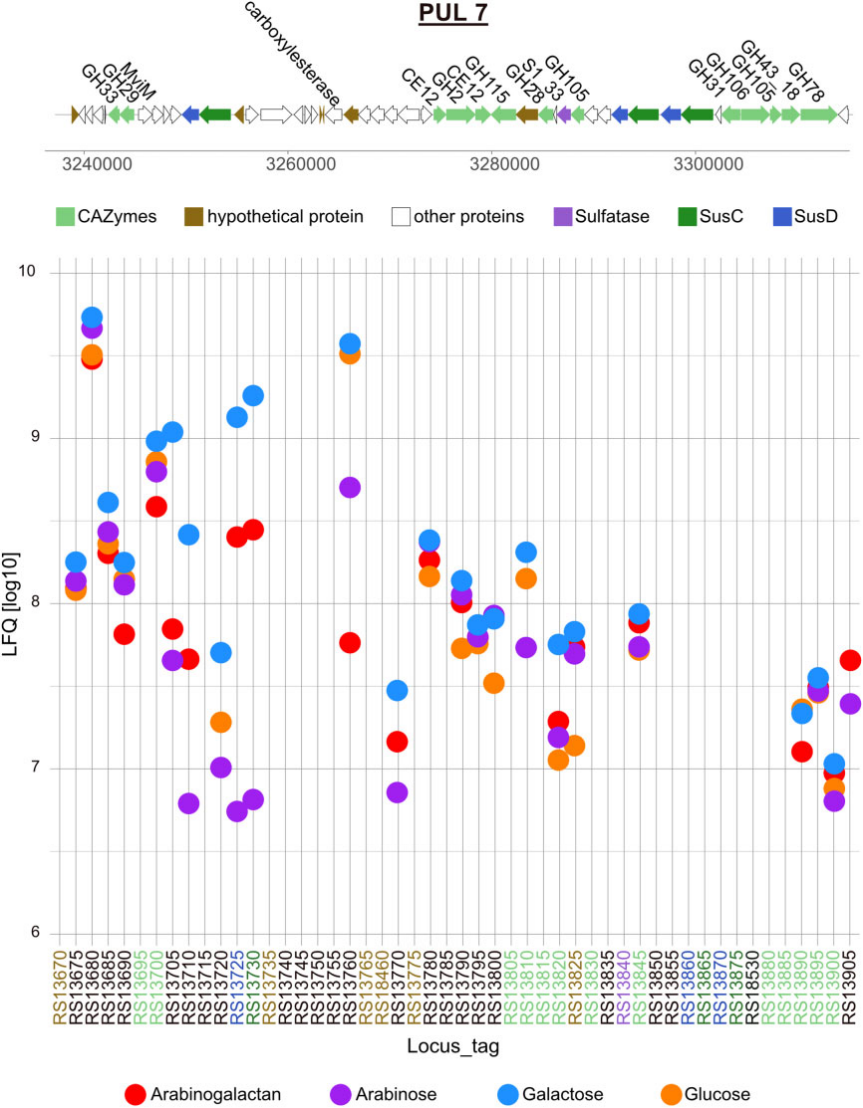
Gene organization and expression of polysaccharide utilization locus 7 of *Maribacter* sp. MAR_2009_72 grown in the presence of arabinogalactan, arabinose, galactose, and glucose. Expression intensities in the plot are the mean values of three biological replicates of each condition shown in LFQ values [log10].

PUL 7 contains a single SusC/D pair and a tandem of SusC/D pair in one genetic region. It encodes 42 enzymes, 13 being classified as CAZymes, three SusC/D pairs, and one sulfatase (Fig. 5). One SusC/D pair and 6 CAZymes were expressed in arabinogalactan grown cells. SusC (JM81_RS13730) and SusD (JM81_RS13725) were expressed in the galactose and arabinogalactan proteomes 100-fold and 10-fold stronger than in the arabinose proteome, respectively, and not in the glucose proteome. The tandem SusC/D pairs were not detected in any of the proteomes. An α-l-fucosidase of the GH29 family (JM81_RS13700) had the highest expression among the CAZymes in this PUL. The GH29 was expressed in similar intensities in all four conditions, suggesting a constitutive expression of this periplasmic enzyme. Less intense, but also expressed in all proteomes was a xylan-α-1,2-glucuronidase belonging to the GH115 family (JM81_RS13820), with the strongest expression on galactose. Two GH105 unsaturated rhamnogalacturonyl hydrolases (EC 3.2.1.172) (JM81_RS13845 and JM81_RS13890) were expressed in all four growth conditions, with the exception of JM81_RS13890, which was not detected in the arabinose proteome. A GH43_18 (JM81_RS13895) was expressed in all four proteomes with similar expression intensities. An α-l-rhamnosidase GH78 (JM81_RS13900) was expressed under all growth conditions. During our analysis a hypothetical protein (JM81_RS13825) with a six-hairpin GH like family domain sparked our interest. It was expressed in all four proteomes, with higher intensities in arabinogalactan, arabinose, and galactose proteomes.

**Figure 6.**
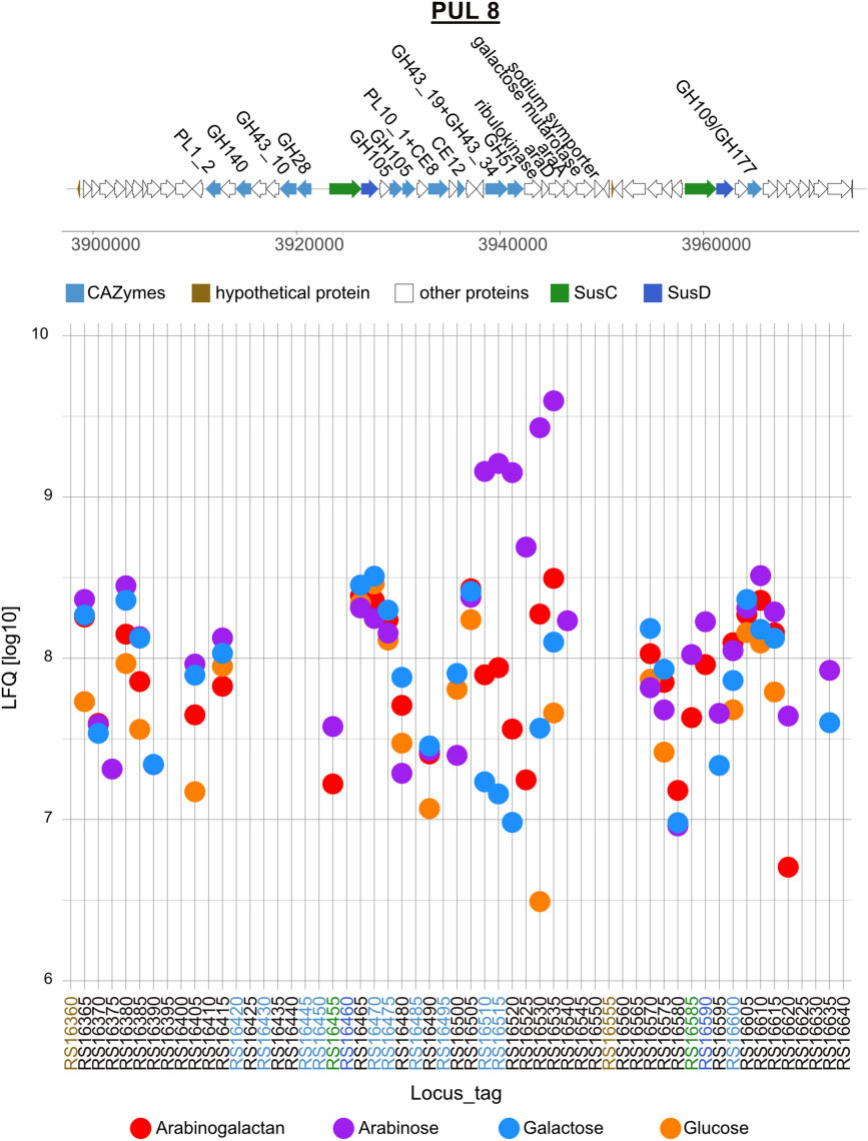
Gene organization and expression of polysaccharide utilization locus 8 of *Maribacter* sp. MAR_2009_72 grown in the presence of arabinogalactan, arabinose, galactose, and glucose. Expression intensities in the plot are the mean values of three biological replicates of each condition shown in LFQ values [log10].

PUL 8 encodes a total of 58 proteins, including 11 CAZymes and two SusC/D pairs (Fig. 6). A total of five CAZymes, two SusCs but only one SusD were expressed in arabinogalactan grown cells. SusC (JM81_RS16585) and SusD (JM81_RS16590) were expressed in the arabinose and arabinogalactan proteome. Another SusC (JM81_RS16455) showed expression, slightly lower than the other SusC, in the arabinose proteome and slightly less for arabinogalactan. Two GH105 proteins (JM81_RS16470 and JM81_RS16475) annotated as unsaturated rhamnogalacturonyl hydrolases were expressed similar in all proteomes. JM81_RS16510 includes two domains, GH43_19 and GH43_34. It was expressed in the arabinose, arabinogalactan, and galactose proteome, whereby the highest intensities were measured for arabinose. Another α-l-arabinofuranosidase, a GH51 (JM81_RS16515), was expressed in a similar pattern to the GH43_19+GH43_34 protein. These two genes are followed by genes of the arabinose metabolism to the pentose phosphate pathway—ribulokinase, l-ribulose-5-phosphate 4-epimerase, and l-arabinose isomerase—and a gene for a galactose mutarotase. All proteins in this operon were expressed in the arabinose, arabinogalactan, and galactose proteome, with highest intensities in arabinose proteomes. Unknown is the function of a GH109, a member of the Gfo/ldh/MocA superfamily of NAD(P) dependent oxidoreductases, that had the highest expression in the arabinogalactan proteome. The expression of a mannonate dehydratase (JM81_RS16615) hinted at a sugar acid metabolism. Interestingly, PUL 8 is preceded by an operon with sugar acid metabolizing enzymes. The following enzymes were induced in the arabinogalactan proteome in comparison to glucose: 5-dehydro-4-deoxy-d-glucuronate isomerase, gluconate-5-dehydrogenase, a sugar kinase, 2-dehydro-3-deoxy-phosphogluconate aldolase, and tagaturonate reductase.

An analysis with dbCAN3 identified 153 CAZymes in the genome, of which 106 were detected in the proteomes. Outside of the PULs 1, 7, and 8, several CAZymes were expressed in arabinogalactan degradation. Many expressed CAZymes had a signal peptide for export out of the cytosol (Table S1 and Fig. S2, Supporting Information). Three of the CAZymes were annotated as GH family 3 enzymes. JM81_RS00095 was expressed in all four conditions, the highest intensities were measured in the arabinogalactan proteome (Fig. S2A, Supporting Information). The second GH3 (JM81_RS08450) was expressed in all four conditions, but with a three to four times larger expression in arabinogalactan, arabinose, and galactose (Fig. S2B, Supporting Information). A third GH3 (JM81_RS18250) was as well expressed in all four conditions, but the highest intensities were measured for arabinose and galactose. It was part of an operon also including an endo-1,4-β-xylanase (GH10) expressed only in arabinose and galactose grown cells (Fig. S2C, Supporting Information). All three GH3 were annotated as galactosidases. A GH43_26 (JM81_RS08585) was expressed in all four datasets, whereby the highest intensities were recorded for arabinose and nearly identical LFQs for glucose and arabinogalactan (Fig. S2D, Supporting Information). A GH115 xylan-α-1,2-glucuronidase (JM81_RS03245) was only expressed in arabinose and arabinogalactan grown cells (Fig. S2E, Supporting Information).

The transport of the monosaccharides across the inner membrane may be facilitated by an ABC transport system consisting of ABC substrate-binding (JM81_RS03610), ABC permease (JM81_RS16840), and ABC ATP binding proteins (JM81_RS01625).

Marine glycans are often decorated with sulfate. We identified 13 sulfatases in the genome of MAR_2009_72, of which three were expressed in arabinogalactan grown cells. JM81_RS05685, JM81_RS05692, and JM81_RS076760 were equally expressed in all four proteomes. All three were previously affiliated with the utilization of mucin, which contains to some extent galactose (Tailford et al. 2015, Glover et al. 2022).

We also detected expressed proteins of the degradation pathway that where not classified as CAZymes. For the degradation of galactose, a galactose mutarotase (JM81_RS1635), galactokinase (JM81_RS01495), UDP-glucose-4-epimerase (JM81_RS00155), and nucleotidyltransferases (JM81_RS00330/14030/10945) were induced in the arabinogalactan proteome. Rhamnose and glucuronic acid are minor sugars in arabinogalactan from larch. Besides the aforementioned CAZymes and enzymes, three proteins related to the metabolism of sugar acids were induced in the arabinogalactan proteome. Gluconate dehydrogenases JM81_RS04310 and JM81_RS04965 yield 2-dehydro-d-gluconate. A 3-dehydro-l-gulonate 2-dehydrogenase (JM81_RS04965) was also induced.

### CFU microbiomes

North seawater sampled on 28 May 2022, at the beach of Sahlenburg, Cuxhaven, Germany, (N53.863607°, E8.593004°) yielded after 12 days of incubation a CFU microbiome of 2.57 × 10^4^/ml on arabinogalactan and of 2.32 × 10^4^/ml on ZoBell’s 2216 marine agar. Partial 16S rRNA gene sequences of abundant CFU on arabinogalactan revealed 27 different genera within 88 colonies, with *Algoriphagus, Leeuwenhoekiella, Maribacter, Sulfitobacter, Yoonia-Loktanella*, and *Pseudoalteromonas* being present with 6–13 CFU. Only two of 63 CFU on ZoBell’s 2216 marine agar, but 29 of 88 CFU on arabinogalactan plates belonged to *Flavobacteriaceae*. Additionally, we investigated autumn samples. Seawater sampled 18–21 October 2022 on Sylt, Germany (N55.031417°, E8.441802°), showed after 18 days of incubation 5.06 (+/−0.88) × 10^5^ CFU/ml for glucose and of 8.32 (+/− 2.21) × 10^5^ CFU/ml for arabinogalactan (*n* = 2). At the sandy west beach (N55.037505°, E8.383341°) plating of pore water yielded 1.75 (+/−1.30) × 10^6^ CFU/ml on for glucose and 0.90 (+/− 0.64) × 10^6^ CFU/ml for arabinogalactan (*n* = 3).

## Discussion

Galactose belongs to the four abundant monosaccharides in planktonic organic matter, mainly as part of polysaccharides and more complex molecules, i.e. arabinogalactan proteins. Plating sea and sediment pore water on arabinogalactan medium showed a large microbiome with the capacity to utilize arabinogalactan for growth. Together with the recent finding that particle-associated bacteria dominate the readily culturable fraction of seater microbiomes (Heins and Harder 2023) this observation indicates that arabinogalactan is a common carbon source for particle-associated bacteria.

Arabinogalactan degradation pathways were so far only described for bacteria from gut and plant systems, but not for marine bacteria (Shulami et al. 2011, Ndeh et al. 2017, Cartmell et al. 2018, Luis et al. 2018, Fujita et al. 2019, Wang and LaPointe 2020, Sasaki et al. 2021). These studies provided information regarding enzymes involved in arabinogalactan utilization, which includes GH families GH43, GH51, GH27, and GH28, often organized in PULs (Shulami et al. 2011, Cartmell et al. 2018, Luis et al. 2018). Hence, we inspected first the upregulated proteins in arabinogalactan grown cells in comparison to glucose grown cells. After a discussion of the SusC/D systems, we analyzed the uniqueness of marine PULs for arabinogalactan degradation in *Maribacter* sp. MAR_2009_72.

Enzymes annotated as members of the GH family 43 are most frequently linked with arabinogalactan utilization. This protein family is one of the largest GH families and has currently 39 subfamilies, whereby only a part has been characterized enzymatically. Most common enzymatic functions are β-d-xylosidase (3.2.1.37), α-l-arabinofuranosidase (3.2.1.55), endo-α-l-arabinonase (3.2.1.99), and 1,3-β-galactosidase (3.2.1.145) (Mewis et al. 2016), with no correlation between subfamily classification and enzyme activity (Kelly 1999, Wang and LaPointe 2020), but as common feature the presence of three active sites in the enzymes. Still, many need a biochemical characterization (Pons et al. 2004, Mewis et al. 2016). The genome of *Maribacter* sp. MAR_2009_72 encodes five GH43 and four of these were shown to be expressed in this study. Subfamilies GH43_1 (JM81_RS00875) and GH43_26 (JM81_RS08585) are characterized so far solely as α-l-arabinofuranosidases. They hydrolyze via retention of configuration (Pitson et al. 1996) the terminal nonreducing residues in α-l-arabinoside side chains of hemicelluloses, like arabinoxylan, arabinogalactan, and arabinan (Shallom et al. 2002a, b). JM81_RS16510 encodes a protein that consists out of two GH43 domains, GH43_19 and GH43_34. Over 70% of subfamily GH43_34 proteins contain a second GH43 domain (Mewis et al. 2016). GH43_34 domains are usually located at the C-terminus and considered to be membrane-spanning (Mewis et al. 2016). The function is not clear, but they may be involved in carbohydrate binding (Mewis et al. 2016). Alike the GH43_1 and GH43_26 proteins, the GH43_19 domain is annotated as a catalytic α-l-arabinofuranosidase domain. The fourth GH43 protein is a member of subfamily GH43_18 (JM81_RS13895), which mostly includes α-l-arabinofuranosidases.

The α-l-arabinofuranosidases function is also annotated to GH51 proteins, although the amino acid sequence differs by large from GH43 enzymes (Sevim et al. 2017). The acid/base catalyst of GH51 is E176 in *Thermobacillus xylanticus* and E175 in *Geobacillus stearothermophilus* T-6 (Debeche et al. 2002, Shallom et al. 2002a). The GH51 from MAR_2009_72 (JM81_RS16515) has an E177, which aligns perfectly with the catalytic glutamic acid of *T. xylanticus* and *G. stearothermophilus* T-6. The amino acid E294 in *G. stearothermophilus* T-6 was identified as catalytic nucleophile (Shallom et al. 2002b), this glutamate is also conserved in GH51 of MAR_2009_72. In contrast to the inverting α-l-arabinofuranosidases of family GH43, α-l-arabinofuranosidases of GH51 hydrolyze bonds retaining the anomeric configuration (Pitson et al. 1996).

The family GH105 comprises unsaturated rhamnogalacturonyl hydrolases, which have been suggested to remove l-rhamnose-α-1,4-d-galacturonic acid on arabinogalactan (Munoz-Munoz et al. 2021). Four GH105 proteins were expressed. The catalysis involves hydrolysis of bonds through syn-hydration of double bonds between C4 and C5 carbons of enopyranuronosyl residues of substrates (Itoh et al. 2006a, b). The catalytic aspartate side chain D143 of GH105 is conserved in GH105 proteins of MAR_2009_72 (Itoh et al. 2006a).

The transport of the oligosaccharides involved several SusC/D pairs. PULs 1, 7, and 8 encode the three SusC/D systems that had the highest expression intensities of all SusC/Ds in the arabinogalactan proteome. On the basis of the dedicated substrate specificity of SusC/D transport systems, we propose two explanations for the induction of several SusC/D pairs: (i) the extracellular hydrolysis of larch wood arabinogalactan generates a mixture of structurally different oligosaccharides which need dedicated transport system and (ii) a signal molecule derived from larch wood arabinogalactan may induce the expression of proteins that may not be necessary for larch wood arabinogalactan, but for the degradation of marine arabinogalactans. The structural diversity of arabinogalactans in terrestrial system is well characterized (Fujita et al. 2019, Villa-Rivera et al. 2021, Leszczuk et al. 2023), but marine arabinogalactans are understudied.

In the periplasm the oligosaccharides are further hydrolyzed by a range of CAZymes. Some PULs (1 and 7) expressed enzymes that can generate monomers. Furthermore, the proteome detected CAZymes that are not encoded in PULs and are predicted to be periplasmatic. The GH10 of PUL 1 was annotated as an endo-1,4-β-xylanase, which indicates that arabinoxylans may also be a substrate for the enzymes of PUL 1. The expression of an α-glucuronidase annotated to GH67, coincides with the presence of glucuronic acid in side chains of arabinogalactan. GH67 removes glucuronic acid from side chains by a single displacement mechanism using an inverting mechanism (Shulami et al. 1999, Biely et al. 2000, Nagy et al. 2002). But it only removes glucuronic acid from nonreducing ends of the oligo- and polysaccharides. A broader substrate range is known for GH115 proteins, which remove glucuronic acid from terminal and internal regions of oligosaccharides (Ryabova et al. 2009, Aalbers et al. 2015). The presence of both GH families, GH67 and two GH115, suggests that glucuronic acid is part of the decoration of arabinogalactans. The expression of the GH29 argues for fucose as a decorating sugar. Enzymes of the family GH29 are exo-α-fucosidases and cleave via an retaining mechanism (Grootaert et al. 2020). Also, rhamnose as specific substrate is supported by expression of a GH78, α-l-rhamnosidase. This GH family solely includes rhamnosidases, which use an inverting mechanism to hydrolyze bonds in cooperation with their catalytic residues (Cui et al. 2007). The galactan backbone hydrolysis requires a β-d-galactosidase. This enzymatic function is frequent among members of the GH family GH3. The proteome detected three expressed GH3 proteins. Final steps of the arabinogalactan pathway include the translocation through the inner membrane, likely via an ABC transport system, and cytoplasmic transformations to channel galactose, arabinose, glucuronic acid, rhamnose, and fucose into the pentose phosphate pathway and the glycolysis.

We investigated the distribution of PUL 1, 7, and 8 of *Maribacter* sp. MAR_2009_72 in the PULDB database using the expressed CAZymes (Terrapon et al. 2018). Homologs of PUL 1 have been characterized for human gut bacteria and *Bacteroides* spp. for the utilization of a range of xylan polysaccharides including arabinoxylan (Martens et al. 2008, Rogowski et al. 2015, Wang et al. 2016). The PUL was *in silico* detected in genomes of a large variety of *Bacteroidota*. In contrast, PUL 7 has so far not been studied experimentally. An *in silico* search detected a homologous PUL structure in *Maribacter sedimenticola* DSM19840 (Nedashkovskaya et al. 2004). PUL 8 has also a homolog in *M. sedimenticola* DSM19840 and other *Bacteroidota*.

A recent metagenomic study of particle-associated bacteria detected a GH43-rich PUL in a *Maribacter* MAG, which the authors annotated as an arabinogalactan PUL (Wang et al. 2024). This PUL is different to the PULs we identified for arabinogalactan in the genome of *Maribacter* sp. MAR_2009_72.

Our observations revealed a substrate specificity of the three PULs. In PUL 1, arabinogalactan is the only inducer for SusC/D, and the expression of a glucuronidase and a xylanase suggests that also glycans with these sugars are substrates for the PUL (Fig. S3, Supporting Information). This hypothesis is supported by previous studies with gut bacteria (Martens et al. 2008, Rogowski et al. 2015, Wang et al. 2016). PULs 7 and 8 have so far not been experimentally observed. PUL 7 is characterized by a very strong induction of SusC/D by galactose and arabinogalactan (Fig. S4, Supporting Information). Galactose is for several proteins the strongest inducer, suggesting galactans as substrate. The presence of fucosidase, glucuronidase, and rhamnosidase suggests a decoration of the marine galactans with the corresponding monosaccharides. PUL 8 is dedicated to arabinose containing glycans. The SusC/D is induced by arabinose and arabinogalactan (Fig. S5, Supporting Information). Besides GHs, the genetic region of PUL 8 includes also monosaccharide-transforming cytoplasmatic enzymes for arabinose and sugar acids. This PUL shows that the consideration of cytosolic carbohydrate-transforming enzymes in the bioinformatic analysis of PULs may improve predictions of substrate specificity.

**Figure 7.**
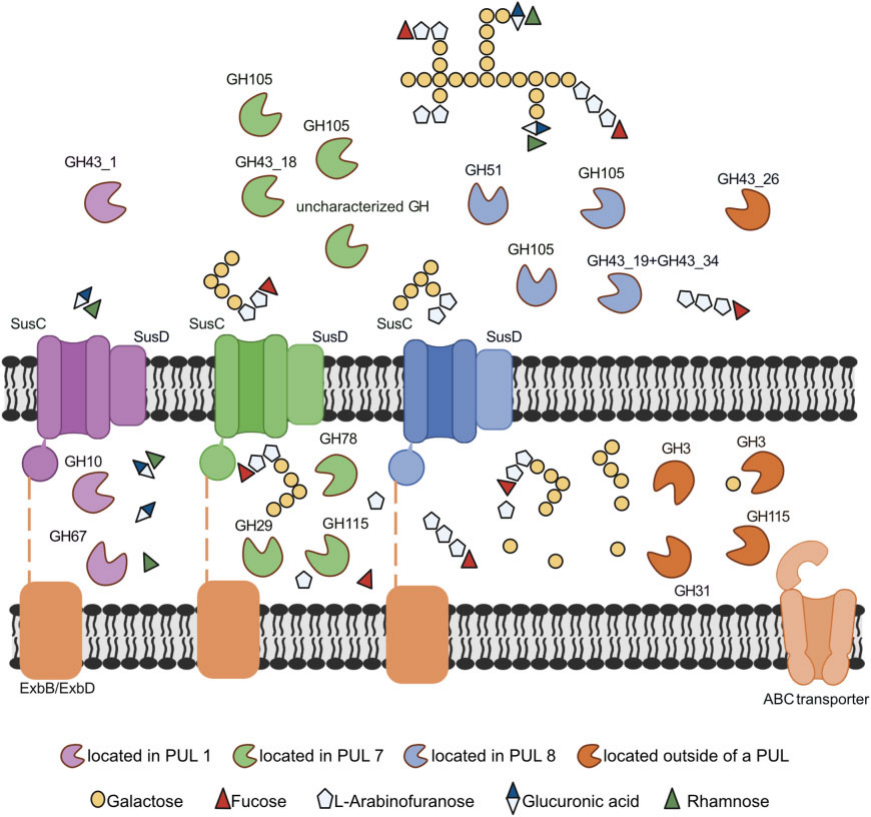
Proposed utilization pathway for arabinogalactan by *Maribacter* sp. MAR_2009_72. Poly- and oligosaccharide structures are not experimentally verified. PUL 1: GH43_1 (JM81_RS00875), SusC (JM81_RS00910), SusD (JM81_RS00905), GH10 (JM81_RS00885), and GH67 (JM81_RS00895). PUL 7: GH105 (JM81_RS13845), GH105 (JM81_RS13890), GH43_18 (JM81_RS13895), uncharacterized GH (JM81_RS13825), SusC (JM81_RS13730), SusD (JM81_RS13725), GH29 (JM81_RS13700), GH78 (JM81_RS13900), and GH115 (JM81_RS13820). PUL 8: GH105 (JM81_RS16475), GH105 (JM81_RS16470), GH51 (JM81_RS16515), GH43_19+GH43_34 (JM81_RS16510), SusC (JM81_RS16585), and SusD (JM81_RS16470). Outside of the PULs: GH43_26 (JM81_RS08590), GH3 (JM81_RS00095), GH3 (JM81_RS08450), GH31 (JM81_RS06015), CBM48+GH13_9 (JM81_RS06020), GH115 (JM81_RS03245), ExbB (JM81_RS07935), ExbD (JM81_RS07930), ABC substrate-binding (JM81_RS03610), ABC permease (JM81_RS16840), and ABC ATP binding (JM81_RS01625). The figure was created with BioRender.com.

The comparative proteomic analysis of larch wood arabinogalactan degradation by *Maribacter* sp. MAR_2009_72 identified expressed proteins encoded in three PULs and outside of PULs (Fig. 3). In summary, members of the GH families 43, 51, and 105 may produce a variety of oligosaccharides. At least three SusC/D systems are involved in the transport into the periplasm, where enzymes belonging to the GH families 3, 10, 29, 67, 78, and 115 produce monosaccharides. The interplay of all these enzymes allows for the utilization of arabinogalactan, which we have summarized in a graph (Fig. 7). The plant polysaccharide structure is expected to be less complex than the variety of arabinogalactans present in the marine habitat (Pfeifer et al. 2020). This may explain why not all CAZymes of each PUL were detected as expressed proteins. A difference between this study of a marine bacterium and previous studies on gut and plant associated bacteria was the presence of GH105 enzymes and the absence of GH27 and GH28 enzymes. Future studies might characterize marine arabinogalactans and enzymatic studies will resolve the individual functions of the induced proteins to provide further information on the microbial utilization.

## Supplementary Material

fiae045_Supplemental_Files
