# Supplementary material for: Proteomic insight into arabinogalactan utilization by particle-associated Maribacter sp. MAR_2009_72
Source: FEMS Microbiol Ecol. 2024 Apr 3;100(5):fiae045. doi: 10.1093/femsec/fiae045 (PMC11036162; doi:10.1093/femsec/fiae045)
Supplement: fiae045_Supplemental_Files [file fiae045_supplemental_files.zip › Supplement Material.docx]

**Supplementary Figures**


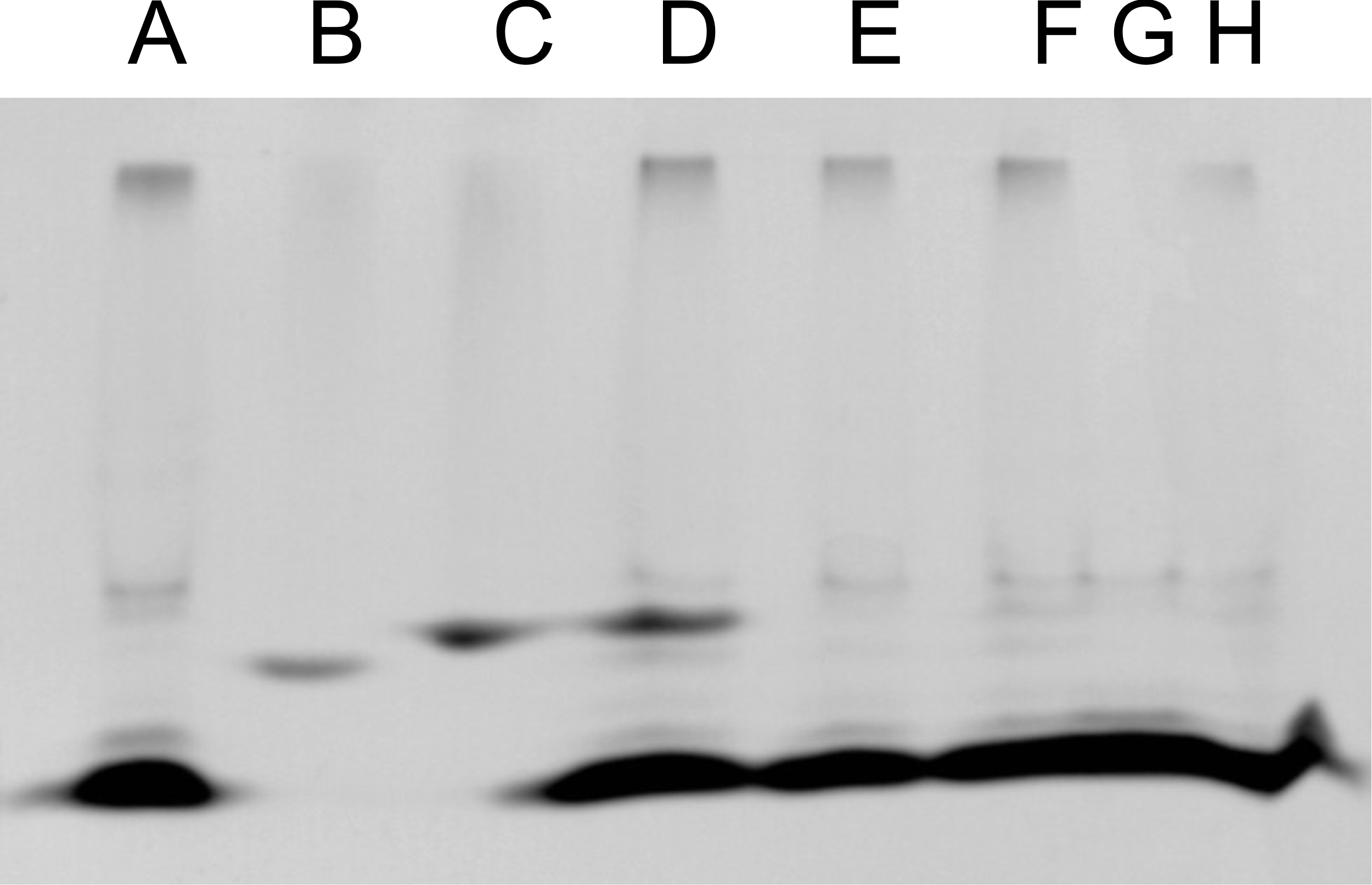


**Supplement Figure 1**: Fluorescently labelled carbohydrates separated by size in an acrylamide gel. A 2 µl of 2 g/L arabinogalactan; B 2 µl of 0.2 g/L arabinose; C 2 µl of 0.2 g/L galactose; D arabinogalactan degrading reaction catalyzed by soluble protein fraction; E arabinogalactan degrading reaction catalyzed by membrane protein fraction; F arabinogalactan degrading reaction catalyzed by heat-inactivated soluble protein fraction; G reaction D lacking arabinogalactan; H reaction D lacking the soluble protein fraction

Methods

Cells grown on arabinogalactan to the late logarithmic growth phase (OD 0.2 – 0.3) were harvested by centrifugation at 16000x *g* for 15 min at 21°C. The cell pellet was transiently stored at -20°C. Cells were lyzed in 1 mL 50 mM 3-(N-morpholino) propanesulfonic acid (MOPS), 2 mM dithiothreitol, pH 7.0 by sonification on ice using a Sonoplus HD70 Bandelin MS73 (BANDELIN, Berlin, Deutschland) with a titanium sonotrode MS73 (BANDELIN) for 4 min at 50% power and 50% cycle (0.5 sec on, 0.5 sec off). The soluble protein fraction was separated by centrifugation for 15 min at 16000x *g* at 21°C. The pellet was resuspended in 1 mL MOPS buffer and named membrane protein fraction. Protein concentration was determined by the Bradford protein assay using bovine serum albumin as standard (Bradford, 1976). Arabinogalactan assays were performed with 11 and 8 µg protein for the soluble and the membrane protein fraction, respectively, and 250 µg arabinogalactan in 100 µl MOPS for 20 h at 21°C. After vacuum drying at 45°C for 1 hour (Eppendorf Concentrator plus, Eppendorf, Hamburg, Germany), the sugars were labelled with 2 µl of 0.2 M 8-amino-1,3,6-naphthalenetrisulfonate (ANTS) in the presence of 5 µl 1M cyanoborohydride at 37°C for 20 h. Then the reaction was diluted with 25 µl 25% vol/vol glycerol and analyzed on 30% v/v acrylamide gels following a protocol for fluorophore-assisted carbohydrate electrophoresis (FACE) (Becker *et al.*, 2017). We introduced a concentration of 65 mM sodium chloride into the separation gel, which allowed a separation of hexose (galactose) and pentose (arabinose). Separation was performed at 100 V for 30 minutes, followed by 200 V for 60 minutes. Gels were documented using a Bio-Rad GelDoc EZ Gel Imaging System (Cambridge Scientifix, Watertown, US).


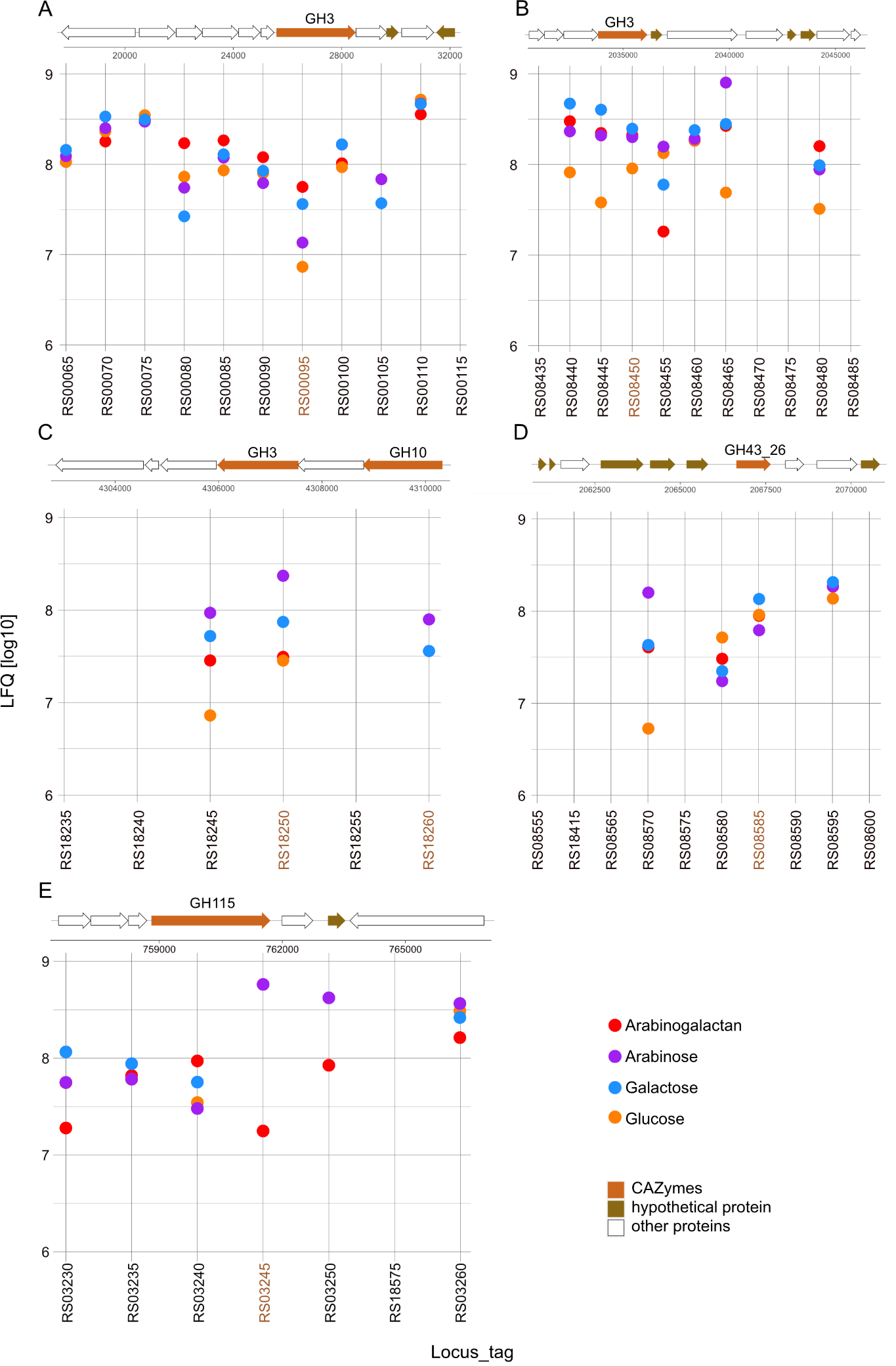


**Supplement Figure 2**: Gene organization and expression of CAZymes located outside of polysaccharide utilization loci of *Maribacter* sp. MAR_2009_72 grown in the presence of arabinogalactan, arabinose, galactose and glucose. Expression intensities in the plot are the mean values of three biological replicates of each condition shown in LFQ values [log10]. A: GH3 (JM81_RS00095) α-galactosidase; B: GH3 (JM81_RS08450) α-galactosidase; C: GH3 (JM81_RS18250) α-galactosidase, GH10 (JM81_RS18260) endo-1,4-β-xylanase; D: GH43_26 (JM81_RS08585) α-L-arabinofuranosidase; E: GH115 (JM81_RS03245) xylan-α-1,2-glucuronidase


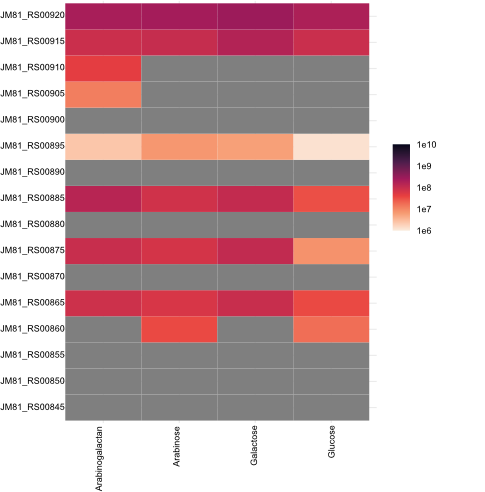


**Supplement Figure 3:** Heatmap of polysaccharide utilization locus 1 of *Maribacter* sp. MAR_2009_72 grown in the presence of arabinogalactan, arabinose, galactose and glucose. Expression intensities in the plot are mean values of three biological replicates of each condition shown in LFQ values [log10].


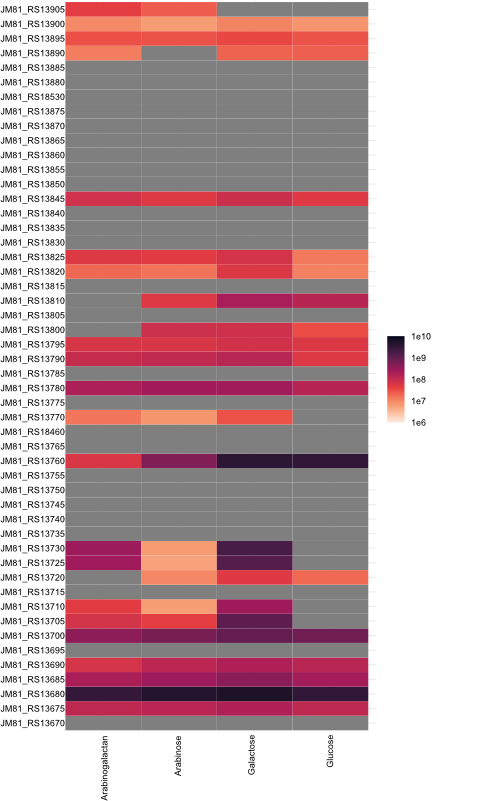


**Supplement Figure 4:** Heatmap of polysaccharide utilization locus 7 of *Maribacter* sp. MAR_2009_72 grown in the presence of arabinogalactan, arabinose, galactose and glucose. Expression intensities in the plot are mean values of three biological replicates of each condition shown in LFQ values [log10].


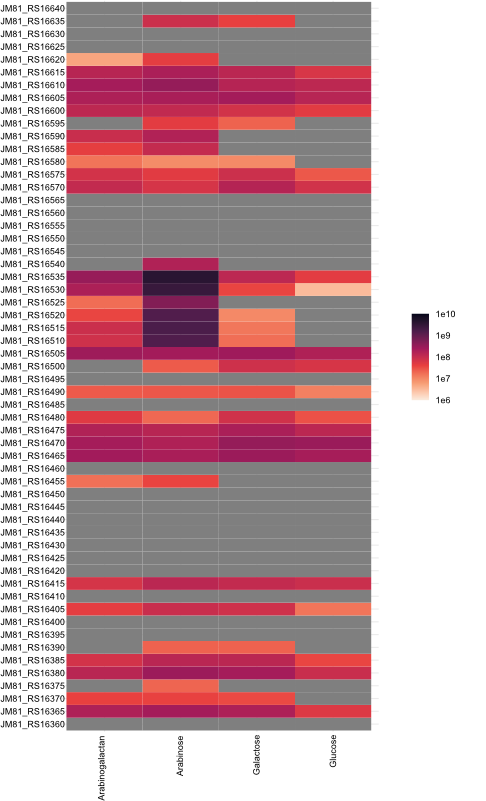


**Supplement Figure 5:** Heatmap of polysaccharide utilization locus 8 of *Maribacter* sp. MAR_2009_72 grown in the presence of arabinogalactan, arabinose, galactose and glucose. Expression intensities in the plot are mean values of three biological replicates of each condition shown in LFQ values [log10].

**References**

Becker, S., Scheffel, A., Polz, M.F., and Hehemann, J.-H. (2017) Accurate quantification of laminarin in marine organic matter with enzymes from marine microbes. *Applied and Environmental Microbiology* **83**: e03389-03316.

Bradford, M.M. (1976) A rapid and sensitive method for the quantitation of microgram quantities of protein utilizing the principle of protein-dye binding. *Analytical Biochemistry* **72**: 248-254.
